# Rumination in posttraumatic stress and growth after a natural disaster: a model from northern Chile 2014 earthquakes

**DOI:** 10.3402/ejpt.v7.31638

**Published:** 2016-11-28

**Authors:** Francisco Leal-Soto, Marcos Carmona-Halty, Rodrigo Ferrer-Urbina

**Affiliations:** 1 Department of Social Sciences, Universidad de Tarapacá, Iquique, Chile; 2 School of Psychology & Philosophy, Universidad de Tarapacá, Arica, Chile

**Keywords:** Natural disaster, basic beliefs, social share of emotion, tsunami, subjective severity

## Abstract

**Background:**

Traumatic experiences, such as natural disasters, produce multiple and serious impacts on people. Despite the traditional focus on negative consequences, in many cases there are also positive consequences, such as posttraumatic growth. Tedeschi and Calhoun proposed a model of posttraumatic growth that emphasizes the role of rumination after the basic beliefs breakdown due to the occurrence of a traumatic experience.

**Method:**

A total of 238 volunteers affected by two major earthquakes and tsunami alerts in northern Chile on April 1 and 2, 2014, responded to an online survey measuring subjective severity, basic beliefs change, social share of emotion, rumination, posttraumatic stress, and posttraumatic growth.

**Results:**

Path analyses reveal that posttraumatic stress goes through a negative change in basic beliefs, intrusive rumination, and deliberated rumination, meanwhile posttraumatic growth is only achieved directly from a positive change in basic beliefs and deliberated rumination.

**Discussion:**

The model is consistent with the empirical model obtained in Chilean people affected by the earthquake and tsunami that occurred on 27 February, 2010, but it is slightly different and in a form that is more consistent with Tedeschi and Calhoun’s theoretical model. Both models remark on the role of deliberated rumination in posttraumatic growth and failure to progress from intrusive to deliberated rumination in posttraumatic stress, but the proposed one is more parsimonious and assumes subjective severity as an antecedent to basic belief changes. These conclusions must be considered in light of limitations that a cross-sectional design and the correlational nature of the statistical analysis carried out impose.

**Highlights of the article:**

- Role of subjective severity, change of basic beliefs, social sharing of emotion, and rumination on posttraumatic stress and growth were modeled from responses of people affected by the April 1–2, 2014, northern Chilean earthquakes.
- Posttraumatic stress goes through negative changes in basic beliefs, intrusive rumination, and deliberated rumination.
- Posttraumatic growth is achieved from positive changes in basic beliefs and deliberated rumination.
- Deliberated rumination and moving from intrusive to deliberated rumination appear as cornerstones in posttraumatic processing.

The Chilean people have moved from one natural disaster to another, particularly related to seismic events. This has allowed its population to develop individual and collective strategies for coping that have been constructed over time. For this reason, Chileans are a particularly interesting group to study regarding the reactions to natural disasters, looking for better ways to promote future coping. In 2010, a major earthquake and tsunami (known as 27-F) struck the central coast. This was one of the longest and most intense earthquakes ever recorded on the planet and had serious consequences, both on human and material (Leiva-Bianchi & Gallardo, 2013). It made evident the vulnerability of the population, causing a general state of alert and lasting apprehension throughout the country, and not only in the affected area. In this context, the northern part of the country suffered two earthquakes of high intensity (8.2° and 7.6° on the Richter scale of seismological magnitude) on April 1 and 2, 2014, whose epicenters were located on the coast, leading to tsunami warnings that forced mass evacuations on consecutive days and caused great upheavals. How were these traumatic events processed by people who lived it? What lessons can we learn to improve the approach to future disaster situations?

Although natural disasters have multiple impacts on those who live through them (Vázquez, Castilla, & Hervás, 2009), García (2011) noted that the proportion of people developing psychopathological disorders as a result thereof is much lower than the proportion of people that do not, and Calhoun and Tedeschi (2006) suggest that up to 80% or more of those affected by traumatic events report positive effects on personal areas of life. Traumatic experiences or loss can trigger positive psychological changes, which have been conceptualized as posttraumatic growth (Tedeschi & Calhoun, 2004). Growth may even coexist with processes of personal depreciation (Baker, Kelly, Calhoun, Cann, & Tedeschi, 2008; Cann, Calhoun, Tedeschi, & Solomon, 2010). How a traumatic event, such as a natural disaster, can lead to stress or growth is the subject of intense research, and it has been found that posttraumatic growth is positively related to social support and spirituality (Cadell & Regehr, 2003), psychological well-being, physical health, optimism, religiosity, and coping strategies (Helgeson, Reynolds, & Tomich, 2006) and sociodemographic and psychosocial variables (Tsai, Sippel, Mota, Southwick, & Pietrzak, 2016). On the other hand, posttraumatic stress has been linked to maladaptive coping strategies (Cofini, Carbonelli, Cecilia, Binkin, & Di Orio, 2015), depressive symptoms (Dell’Osso et al., 2014), physiological markers (Orr & Roth, 2000), and sociodemographic and psychosocial variables (Chen et al., 2016; Fan, Long, Zhou, Zheng, & Liu, 2015; Flores, Carnero, & Bayer, 2014; Kun, Tong, Liu, Pei, & Luo, 2013).

The subjective severity attributed to the event is associated with both the emergence of stress and the emergence of growth, possibly because, paradoxically, the greatest severity tends to mobilize the person deeper, promoting processes that can lead to development. In this regard, it has been argued that the growth process is triggered when the event is sufficiently severe and basic beliefs about the world are challenged, necessitating their revision or reconstruction (Calhoun, Tedeschi, Cann, & Hanks, 2010; Vera, Carbelo, & Vecina, 2006). The basic beliefs are conceptual schemes, stable cognitive representations about ourselves, others and the world, which allow us to develop expectations that organize and facilitate our sense of control over life (Janoff-Bulman, 1992). Those who face traumatic situations can view these beliefs as being challenged, initiating cognitive processes such as resignification or a search for meaning, which allows them to reconstruct their basic beliefs (Arnoso, Bilbao, & Páez, 2011; Cann et al., 2009; Lindstrom, Cann, Calhoun, & Tedeschi, 2013). The severity of situations experienced can play a role, and it has been demonstrated that extremely negative events can produce a greater positive change in the eudemonic welfare (discovery or development of personal strengths and support from others) that is not necessarily found in events having a negative medium intensity or even positive life events (Bilbao, Páez, Da Costa, & Martínez-Zelaya, 2013). The extreme negative events would most likely lead to the growth by a process that Janoff-Bulman (2004, p. 32) calls “existential reevaluation.”

Changes in basic beliefs can have positive valence, for example, allowing people to see others more positively, constituting a positive change or increased invulnerability, or in contrast, they can have negative valence, for example, causing one to lose confidence in life, constituting a negative change or increased vulnerability (Arnoso et al., 2011). Both types of changes can coexist in response to the same event.

Among the factors that could tilt one toward growth in terms of cognitive elaborations, rumination plays a major role. Rumination is the phenomenon of thinking over and over again in the same matter, in this case, on the traumatic event and elements related to it. Rumination can occur in an unintentional, unwanted, or uncontrollable manner by the person who is flooded with thoughts of the event regardless of his or her will (intrusive rumination, destructive), or it can be deliberately produced by the person in an attempt to understand or make sense of what happened (deliberate rumination, constructive). It has been suggested that intrusive rumination predominates in the early posttraumatic period, leading progressively to elaborate rumination, which would eventually lead to growth; the failure to move from intrusive rumination to deliberate rumination would be one way to develop posttraumatic stress disorder. An in-depth explanation about the involved process can be found in Calhoun, Tedeschi, et al.’s (2010) work, as well as in Cann et al.’s (2011) research.

Although studies on the precedent factors of posttraumatic growth remain inconclusive (Ramos & Leal, 2013), among the variables that have been found to be related to growth after a traumatic situation, in addition to the breakdown of basic beliefs and rumination, are socially sharing the emotional experience (Páez, Basabe, Ubillos, & Gonzalez-Castro, 2007; Rimé, Páez, Basabe, & Martinez, 2010), coping (Páez, Basabe, Bosco, Fields, & Ubillos, 2011), especially in terms of coping that is focused on the problem (García, Cova, Rincón, Vásquez, & Páez, 2016), and forms of collective coping (Villagrán, Reyes, Wlodarczyk, & Páez, 2014).

On the occasion of 27-F in the center of the coastal area of Chile, García, Jaramillo, Martínez, Valenzuela, and Cova (2014) tested a model of psychological responses to the disaster in a sample of affected college students, based on the general model of posttraumatic growth proposed by Tedeschi and Calhoun (2004), also incorporating posttraumatic stress and posttraumatic growth. Their results showed that stress and growth that were subsequent to trauma rely on distinct processes. Although both begin with the breakdown of basic beliefs associated with the subjective severity of the event, the positive change in the basic beliefs leads to socially sharing the emotions and deliberate rumination, reaching, in this way, toward posttraumatic growth; meanwhile, the predominance of negative changes in basic beliefs leads to intrusive rumination and posttraumatic stress. However, if intrusive rumination evolves and becomes elaborate, processing can lead, in this way, to growth. García, Jaramillo, et al.’s (2014) results provide evidence regarding the role of rumination in posttraumatic growth, particularly on moving from intrusive rumination to deliberate rumination, which would allow for positive cognitive processing of events (Calhoun, Tedeschi, et al., 2010). Similar results were obtained with a different sample of people affected by the same event (García, Cova, Rincón, & Vasquez, 2015). Another study including additional variables, and testing its relationship only with posttraumatic growth and not with stress, showed results that are consistent with previous findings (García et al., 2016). In this study, the subjective severity of the event leads to posttraumatic growth in various ways: directly, through coping that is focused on the problem or social sharing of emotions, and by deliberate rumination after an intrusive rumination occurred.

In this article, we set out to test the model García, Jaramillo, et al. (2014) obtained, which highlights the role of intrusive rumination and deliberate rumination on stress and posttraumatic growth, in a sample of people affected by the earthquakes and tsunami warnings that occurred in northern Chile on April 1 and 2, 2014. However, we note that in this model, the subjective severity of the event and the change in the basic beliefs are considered variables that covary, which, in our view, does not reflect fully the theoretical model that it purports to represent. In this model, the subjective severity is an antecedent to change in the basic beliefs (Tedeschi & Calhoun, 2004) and should be represented as an exogenous variable that influences change in basic beliefs in the model. For this reason, our second objective was to test the modified model in this way, considering the subjective severity of the event as an exogenous variable in relation to changes in basic beliefs (Fig. 1).

**Fig. 1:**
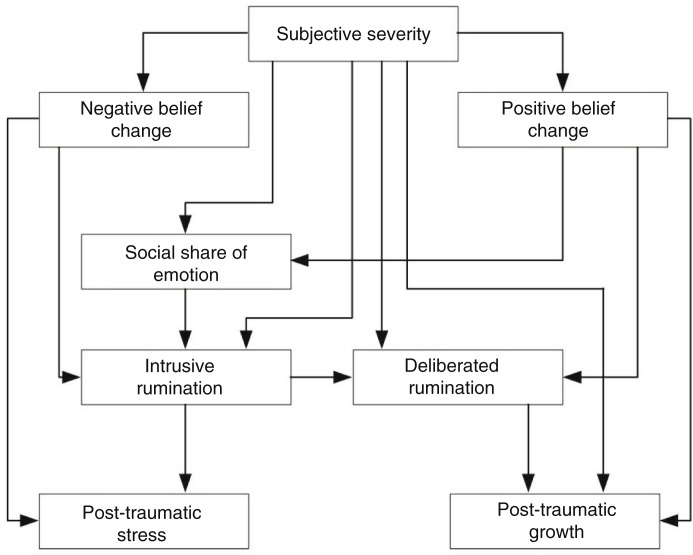
Proposed model.

## Methodology

### Design

The design used was correlational and cross-sectional.

### Participants

The participants were 238 inhabitants of Iquique who lived the events on April 1 and 2, 2014, and agreed to participate in the study voluntarily and without receiving any compensation. Just over half (56%) were women, and the mean age was 29.15 years [standard deviation (SD)=11.5]. Almost half (48.3%) were students attending the same university in which the research began, 15.9% were other members of the university community (faculty, administrative, and service staff) and users of the university’s services center, 26.4% were people linked to some member of the university (family, friends, and acquaintances), and 9.4% were people who had no relationship with the university, but became aware of the survey and completed it. Fifty percent were students, 22.5% were dependent workers, 18.6% were professionals or self-employed, 2.4% were entrepreneurs, and 6.5% were houseworkers or informal workers. Three-quarters of respondents (74.8%) reported having experienced one or more previous catastrophic experiences (earthquake, fire, and traffic accident).

### Measurements and instruments

#### Subjective severity of the event

García, Reyes, and Cova’s (2014) subjective severity of event scale was used. It consists of two questions that assess whether the person perceives the event as being traumatic: “To what degree do you feel your life was altered due to the event?” and “To what degree do you qualify this event as a traumatic experience in your life?” Internal consistency reported by the authors from previous studies is acceptable (Cronbach’s α≥0.69). The survey’s response format used a Likert scale ranging from 0 (*none*) to 4 (*severe*) points.

#### Change in basic beliefs

We used the Impact on Basic Beliefs Questionnaire (IBQ), developed by Corsini and modified by Páez (Bilbao et al., 2013). It consists of six items that measure positive change (e.g., “I did see people more positively”) and six items that measure negative change (e.g., “It made me lose confidence in the other people”). The responses were recorded on a Likert scale with responses ranging from 1 (*completely false*) to 7 (*completely true*). Internal consistency for both subscales was Cronbach’s *α*=0.75 and Cronbach’s *α*=0.72, respectively, as determined in García, Jaramillo, et al.’s (2014) study.

#### Social sharing of emotion

The three questions García, Jaramillo, et al. (2014) used were slightly modified to fit the application time (e.g., the question “During the first six months after the earthquake, did you tell someone what he lived and/or felt that day?” was changed to “I told someone what I lived and/or felt that day”). The response format remained the original Likert scale ranging from 1 (*never*) to 7 (*always*). Internal consistency, as García et al. reported, was Cronbach’s *α*=0.76.

#### Rumination

The Event Related Rumination Inventory (ERRI; Cann et al., 2011) was administered. It measures intrusive rumination (e.g., “I think about what happened unintentionally”) and deliberate rumination (e.g., “I think about whether I can find meaning to what happened”) separately, each with 10 items in a Likert scale format ranging from 0 (*almost never*) to 3 (*almost always*). García, Jaramillo, et al. (2014) translated this scale, and the internal consistency reported by the authors of the original version was Cronbach’s *α*=0.94 for intrusive rumination and Cronbach’s *α*=0.88 for deliberate rumination.

#### Posttraumatic stress

The Impact of Event Scale Revised (IES-R), validated in Chile by Caamaño et al. (2011), was used. It measures three subscales (Avoidance, Intrusion, and Hyperactivation) through 22 items (e.g., “I feel like it had not happened or was not real,” “I think of it, even when I do not want,” and “I am watchful and on guard”) using a Likert scale format ranging from 0 (*none*) to 4 (*extremely*). The internal consistency obtained in the validation sample was Cronbach’s *α*=0.98.

#### Posttraumatic growth

The Spanish version of the Posttraumatic Growth Inventory (PTGI) that Páez et al. (2011) developed was used, which has been validated in Chile (García, Cova, & Melipillán, 2013; Leiva-Bianchi & Araneda, 2015). It is an inventory of 21 items (e.g., “I have more confidence in myself”) using a Likert scale format ranging from 0 (*no change*) to 5 (*a major change*). The internal consistency in García et al.’s (2013) sample was Cronbach’s *α*=0.95.

### Procedure

The research protocol was submitted to and approved by the research ethics committee of the university where the research was conducted. Permission for the administration of the instruments was requested from principals of the university. Questionnaires were loaded onto an online platform. In various ways (posters, announcements in classes and jobs, meetings and personal interviews, and emails), the survey was spread, and members of the university community were invited to participate and were encouraged to invite their relatives and acquaintances; by the same means, the web address and link to the instruments were provided. Data were collected between May and September 2014 over a period of 1–5 months after the events occurred. In the introduction of the survey, explicit references to the April 1 and 2, 2014, earthquakes were made, and all of the subsequent questions were asked in regard to these events.

### Data analysis

The database was built using the statistical package SPSS Statistics, version 22, and the path analyses were performed using the SPSS AMOS, version 21.

## Results

The internal consistency obtained for the scales used was good or acceptable for all measures in this sample: subjective severity, Cronbach’s *α*=0.76; positive change in basic beliefs, Cronbach’s *α*=0.85; negative change in basic beliefs, Cronbach’s *α*=0.81; social sharing of emotion, Cronbach’s *α*=0.79; intrusive rumination, Cronbach’s *α*=0.92; deliberate rumination, Cronbach’s *α*=0.90; posttraumatic stress, Cronbach’s *α*=0.94; and posttraumatic growth, Cronbach’s *α*=0.96. The descriptive statistics of the variables are presented in Table 1.

**Table 1:** Descriptive statistics for variables in model

	Min.	Max.	Mean	Standard deviation
Subjective severity of event (SS)	0	8	2.81	1.78
Negative change in basic beliefs (NBC)	6	35	11.97	6.27
Positive change in basic beliefs (PBC)	6	42	24.73	7.99
Social share of emotion (SSE)	3	21	11.92	3.72
Intrusive rumination (Brooding; BR)	0	28	4.13	4.97
Deliberated rumination (DR)	0	26	4.84	5.14
Posttraumatic stress (PTS)	0	68	10.32	13.24
Posttraumatic growth (PTG)	0	105	31.77	25.91

According to the multivariate kurtosis of Mardia (CR=14.26), it was not possible to sustain the assumption of multivariate normality, so all models in the study were tested using the generalized least squares method, which is more robust compared with the maximum likelihood estimation method in this scenario.

First, adjustment indicators for the three models are presented (Table 2): García, Jaramillo, et al.’s (2014) model with indicators obtained and reported by the authors in their sample, García, Jaramillo, et al.’s (2014) model with indicators estimated using data from the sample of this study, and the proposed model where subjective severity (SS) is an exogenous variable that directly influences the negative change in basic beliefs (NBC) and positive change in basic beliefs (PBC).

**Table 2:** Fit indices for original model (García, Jaramillo, et. al., 2014) and proposed model

Model	No. parameters	χ^2^	DF	*p*	χ^2^/DF	AGFI	TLI	CFI	RMSEA (H_0_: RMSEA=0.05)	SRMR	AIC
1) García et al., original sample	24	19.631	12	0.07*	1.63	**	0.95	0.99	0.047 (*p*=**)	**	**
2) García et al., actual sample	24	45.858	12	0.00	3.821	0.854	0.575	0.818	0.109 (*p=*0.002)	0.0483	93.858
3) Proposed model, actual sample	23	45.858	13	0.00	3.528	0.865	0.619	0.823	0.103 (*p=*0.004)	0.0483	91.858

* Not reported by authors (García, Jaramillo, et. al., 2014), but estimated based on available data;

** not reported by authors (García, Jaramillo, et. al, 2014).

The table illustrates that the adjustment level of García, Jaramillo, et al.’s (2014) original model using the sample of this study is considerably lower compared with what the authors reported using their sample, with more than double the χ^2^ distribution value (Δχ^2^=26.227). In addition, there were more discrepancies between the observed and reproduced matrices in this sample. This results can be attributed both to differences in the estimation methods (García et al. used the maximum likelihood estimation method) and differences in the characteristics of the sample.

**Fig. 2:**
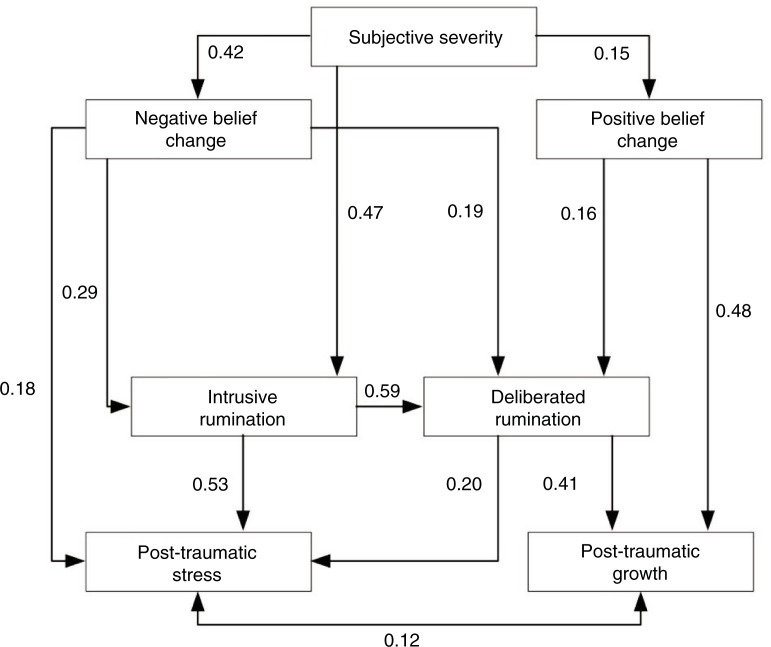
Proposed model respecified (numbers beside arrows represent the standardized direct effects of all the variables in the model and the correlation between PTG and PTS. All of them are statistically significant, *p*<0.05).

Both the original model and the proposed model are below the standards recommended in the literature (Schreiber, Nora, Stage, Barlow, & King, 2006) with the data of this sample, indicating that both models poorly replicate the observed variance–covariance matrix (see Appendix), making it necessary to check for the existence of relationships that are poorly represented by the model. For this purpose, we chose to begin the review from the proposed model, offering a slightly higher adjustment (Δ_AIC_=2.00), which is more parsimonious (Δ_DF_=1) than is the original model; it also, in our opinion, better reflects the overall base model (Tedeschi & Calhoun, 2004). A review of the adequacy of the direct and indirect represented effects consisted of iterated elimination of the effects for which the null hypothesis (H_0_: *r*=0) cannot be rejected (*p*>0.05), and the incorporation of the effects whose residuals are greater than expected by chance with 95% safety (SR>1.96). In addition, the variable social sharing of emotion (SSE) was removed because it did not significantly affect any other variable in the model. These modifications ended up forming a new model (Fig. 2), which corresponds to a respecification of our initial proposal and proved to be a good representation of the sample relations, according to all adjustment indicators (Table 3).

**Table 3:** Fit indices for proposed model respecified

No. parameters	χ^2^	DF	*p*	χ^2^/DF	AGFI	TLI	CFI	RMSEA (H_0_: RMSEA=0.05)	SRMR	AIC
20	15.111	8	0.06	1.889	0.936	0.884	0.956	0.061 (*p*=0.300)	0.0319	55.111

In order to assess the relative importance of each variable, the direct, indirect, and total effects present in the model (Table 4) are given. To interpret the magnitude of the observed effects, the rule that Cohen (1988) suggested was used: small (*r*>0.1), medium (*r*>0.3), and large (*r*>0.5) effect sizes.

**Table 4:** Direct, indirect and total effects in proposed model respecified

	Direct effects					Indirect effects					Total effects				
			
	SS	NBC	PBC	BR	DR	SS	NBC	PBC	BR	DR	SS	NBC	PBC	BR	DR
NBC	0.421					x					0.421				
PBC	0.154	x				x	x				0.154	x			
BR	0.470	0.289	x			0.122	x	x			0.592	0.289	x		
DR	x	0.192	0.157	0.590		0.454	0.171	x	x		0.454	0.362	0.157	0.590	
PTG	x	x	0.480	x	0.411	0.260	0.149	0.064	0.242	x	0.260	0.149	0.545	0.242	0.411
PTS	x	0.184	x	0.531	0.196	0.481	0.224	0.031	0.115	x	0.481	0.409	0.031	0.646	0.196

BR, intrusive rumination (brooding); DR, deliberated rumination; SS, subjective severity; NBC, negative belief change; PBC, positive belief change; PTG, posttraumatic growth; PTS, posttraumatic stress; x, null effect or effect not represented in model.

The model shows that posttraumatic stress is explained by the large overall effect of intrusive rumination, mostly a direct effect; the medium indirect effect of subjective severity; the overall medium effect of negative change in basic beliefs; and a small direct effect of deliberate rumination. Moreover, posttraumatic growth is explained by the large overall effect of positive change in basic beliefs, mainly as a direct effect; medium direct effect of deliberate rumination; and small and indirect effects of subjective severity, intrusive rumination, and negative change in basic beliefs.

## Discussion

The model García, Jaramillo, et al. (2014) proposed to explain the growth and posttraumatic stress in people affected by the 27-F 2010 in the central Chilean coastal area was based on Tedeschi and Calhoun’s (2004) general model, and both emphasize the change in basic beliefs and rumination that lead to growth. The results obtained in our study with people affected by the earthquakes and tsunami warnings in the north of the country confirm both propositions, but with some differences.

First, in García, Jaramillo, et al.’s (2014) model, intrusive rumination is affected by subjective severity and positive change in basic beliefs through the social sharing of emotion, but social sharing was not relevant in the case of our sample, being excluded from the model. The difference between the two results could be explained, at least partially, by the qualitative differences of events and differences in the samples and their eventual behavior. In the first case, the sample consisted exclusively of college students in Concepción, a city that was directly affected by the earthquake of 27-F, but due to its Mediterranean character, the tsunami alert following the event was unnecessary. In the case of our study, although the sample was obtained from a call made within the university, it included a significant proportion of non-student people, in fact more than 50%, giving a much greater heterogeneity. Additionally, natural events from which our research was preceded included two consecutive tsunami warnings that forced mass evacuations to security zones where the evacuated population is concentrated. We who lived through these experiences can bear witness to the intense sharing of emotion that occurred in these circumstances immediately after the events, marking an important difference. This is supported by the data, as the average social sharing of emotions was significantly higher in our sample than in García, Jaramillo, et al.’s (2014; *t*=10.197, DF=496, *p*<0.000). In addition, the range of responses, which began at 0 in García et al.’s sample but had a low score of 3 in ours, implied that no person in our sample reported not sharing emotions with others, in contrast to García et al.’s sample. Thus, the lower level of variability in terms of social sharing of emotion, coupled with the greater heterogeneity of the sample, could be the basis for the fact that this variable was not relevant in our sample.

The main result of García, Jaramillo, et al.’s (2014) study is the empirical confirmation of the role of rumination in the production of posttraumatic growth, one of the central tenets of Tedeschi and Calhoun (2004), in particular, the role played by the deliberate rumination as a critical element to move toward psychological growth (Calhoun, Tedeschi, et al., 2010). Our results partially support García, Jaramillo, et al.’s (2014) because we found evidence of direct effects of positive changes in basic beliefs and deliberate rumination, but did not find evidence of a direct effect of subjective severity.

Additionally, we found a direct effect of deliberate rumination with posttraumatic stress, which was not reported in García, Jaramillo, et al.’s (2014) study. This is not an unexpected fact according to Tedeschi and Calhoun’s (2004) theoretical view, because posttraumatic growth is a perspective related to traumatic experiences, implying reconceptualization or reorganization of it and giving a new meaning to the person’s life; however, as they state, the experience of growth does not necessarily replace the experience of stress, but can coexist. Deliberate rumination, along with enabling psychological growth, probably also contributes to sustaining the stress of the event; the critical point is not that deliberate rumination suppresses the stress, but that the step from intrusive rumination toward deliberate rumination enables psychological growth. Therefore, the model confirmed by our data seems closer to the theoretical model than to García, Jaramillo, et al.’s (2014), but differences between the groups and reactions to different events should not be surprising because it is recognized that both coping with stressful situations and personal growth are complex and open processes and may present multiple alternative paths (Calhoun & Tedeschi, 2004). However, the continued importance that rumination acquired in empirical studies, such as the present one, shows that rumination is a process that cannot be ignored when studying stress and posttraumatic growth or interventions in disaster or high-stress situations. In fact, deliberate rumination as a form of rebuilding the meaning of experience or framing the experience into a new meaning is the focus of narrative techniques in constructivist therapies (Neimeyer, 2001; Neimeyer & Stewart, 2000); this is a possible way in that deliberated rumination can foster personal strength, appreciation of life and spiritual change, facilitate the discovering of new possibilities or the improvement in the relationships with others, as factors of PTG (Calhoun & Tedeschi, 2006).

The role of subjective severity of the event and its impact on the basic beliefs as triggers of posttraumatic growth is another proposal of Tedeschi and Calhoun’s (2004) general model. This proposal is also reflected in García, Jaramillo, et al.’s (2014) model and is clearly supported by the data of our sample. However, although our results confirm these relationships, these are different from those of García et al. in at least two respects. First, because it is more parsimonious (i.e., it includes fewer relationships), for example, in our model the subjective severity shows no significant direct effect on the deliberate rumination or on posttraumatic growth, it relates to them only indirectly by way of change in the basic and/or rumination beliefs, which also seems more consistent with Tedeschi and Calhoun’s (2004) general model. The second way in which our model differs from García, Jaramillo, et al.’s (2014) is the form that subjective severity and changes in basic beliefs, both positive and negative, are treated. García et al. assume that these three variables are exogenous variables; that is, none of them are considered as receiving the causal effect of another variable in the model. In the theoretical model taken as the basis (Calhoun, Cann, et al., 2010; Tedeschi & Calhoun, 2004), subjective severity of the event is not explicitly considered. In that model, the traumatic event occurs and directly acts as a challenge to the basic beliefs; it is assumed that it represents a subjective impact that is strong enough to produce a breakdown of basic beliefs. The subjective severity of the event, then, is implicitly considered as part of the event, constituting a critical dimension that starts the process that leads to stress or growth. We try to collect it in our model, considering the subjective severity as an exogenous variable that impacts basic beliefs, which become the endogenous variables. This claim was supported by the data.

However, in another version of the same model (Calhoun, Tedeschi, et al., 2010), it is proposed that the basic beliefs are not only challenged by the traumatic event, but can interact with it, for example, providing context for it and, therefore, mediating its effect. This could be understood as a distinction between intrinsic, objective severity or extent of the damage caused by the event, and their subjective severity (i.e., the effect the person perceives given his or her particular set of beliefs and other personal variables that could contextualize the real damage). In psychological research, the role of subjective severity has been emphasized because the psychological processes appear to relate to the meaning the person gives the facts rather than to the facts themselves (Ellis, 1961); however, it is recognized that the severity of the damage that actually occurred in a disaster also plays an important role. For example, Leiva-Bianchi and Araneda (2013a) use the severity of the damage to the home as a result of an earthquake to validate a scale of posttraumatic stress, which involves the assumption that more severe damage produces greater psychological effects. The same authors reported a greater proportion of posttraumatic stress in people who were affected by the earthquake and tsunami of 27-F compared with those who were only affected by the earthquake (Leiva-Bianchi & Araneda, 2013b). For their part, Leiva and Quintana (2010) found that people who suffered property losses or who were exposed to the tsunami or its risk on 27-F showed more symptoms of panic attacks than did those who did not suffer loss of property or who were only exposed to the earthquake but not to the tsunami or its risk. Along the same vein, García, Reyes, and Cova (2014) reported that high actual losses are associated with greater positive change in people with high socioeconomic status. However, except for García, Jaramillo, et al.’s (2014) recently mentioned study, we have no knowledge that objective severity or actual extent of damage was explicitly considered as separate variables in a model of stress or posttraumatic growth. Their inclusion could help to understand the role of subjective severity in relation to a change in basic beliefs as initiators of posttraumatic growth.

This study had a specific aim to test and to improve the model presented by García, Jaramillo, et al. (2014), which provides evidence in favor of Tedeschi and Calhoun’s (2004) model of posttraumatic growth. Because of its focus on a specific model, there are many variables that could fit in a model of posttraumatic growth that are neglected in this study, such as sociocultural influences (Calhoun, Cann, et al., 2010) or personal features like lifestyle, altruism, or medical conditions (Tsai et al., 2016), which need further attention and careful procedures for managing it in periods immediately following natural disasters. On the other hand, this study helps to overcome two of the limitations that García, Jaramillo, et al. (2014) recognized in their work. The first breakthrough is the larger heterogeneity in our sample, as fewer than half were college students; the second is that the data were collected much closer to the occurrence of the natural disaster. However, its design was cross-sectional, so that, as in the case of García et al., the hypothesized relationships in our model would respond to a modeling through structural equations and not from a design that allows for establishing temporary or causal relationships, but rather only establishes its plausibility. This is a hard problem because it is difficult to use another design in the case of people affected by natural disasters; however, the use of longitudinal designs, such as Osenbach et al.’s (2014) or Tsai et al.’s (2016) study, could be helpful in addressing this difficulty.

## Appendix

Variance–covariance and correlations matrix

**Table:** Variance–covariance matrix

	SS	NBC	PBC	BR	DR	PTG	PTS
SS	3.070						
NBC	4.583	38.528					
PBC	2.109	3.148	61.107				
BR	5.076	14.804	3.487	23.975			
DR	4.022	15.352	8.815	17.272	25.580		
PTG	11.262	35.557	110.278	39.921	64.658	608.840	
PTS	11.027	43.784	10.640	48.544	43.451	120.075	171.508

**Table:** Correlations matrix

	SS	NBC	PBC	BR	DR	PTG	PTS
SS	1						
NBC	0.421	1					
PBC	0.154	0.065	1				
BR	0.592	0.487	0.091	1			
DR	0.454	0.489	0.223	0.697	1		
PTG	0.260	0.232	0.572	0.330	0.518	1	
PTS	0.481	0.539	0.104	0.757	0.656	0.372	1
